# Measuring inorganic phosphate and intracellular pH in the healthy and hypertrophic cardiomyopathy hearts by in vivo 7T ^31^P-cardiovascular magnetic resonance spectroscopy

**DOI:** 10.1186/s12968-019-0529-4

**Published:** 2019-03-14

**Authors:** Ladislav Valkovič, William T. Clarke, Albrecht I. Schmid, Betty Raman, Jane Ellis, Hugh Watkins, Matthew D. Robson, Stefan Neubauer, Christopher T. Rodgers

**Affiliations:** 1 Oxford Centre for Clinical Magnetic Resonance Research (OCMR), Division of Cardiovascular Medicine, BHF Centre of Research Excellence, University of Oxford, Oxford, UK; 2 Department of Imaging Methods, Institute of Measurement Science, Slovak Academy of Sciences, Bratislava, Slovakia; 3 Wellcome Centre for Integrative Neuroimaging, FMRIB, Nuffield Department of Clinical Neurosciences, University of Oxford, Oxford, UK; 4 High-Field MR Centre, Centre for Medical Physics and Biomedical Engineering, Medical University of Vienna, Vienna, Austria; 5 The Wolfson Brain Imaging Centre, University of Cambridge, Cambridge Biomedical Campus, Cambridge, UK

**Keywords:** ^31^P CMRS, 7T, Cardiac intracellular pH, 3D-CSI, Cardiac Pi

## Abstract

**Background:**

Cardiovascular phosphorus MR spectroscopy (^31^P-CMRS) is a powerful tool for probing energetics in the human heart, through quantification of phosphocreatine (PCr) to adenosine triphosphate (ATP) ratio. In principle, ^31^P-CMRS can also measure cardiac intracellular pH (pH_i_) and the free energy of ATP hydrolysis (ΔG_ATP_). However, these require determination of the inorganic phosphate (Pi) signal frequency and amplitude that are currently not robustly accessible because blood signals often obscure the Pi resonance.

Typical cardiac ^31^P-CMRS protocols use low (e.g. 30°) flip-angles and short repetition time (TR) to maximise signal-to-noise ratio (SNR) within hardware limits. Unfortunately, this causes saturation of Pi with negligible saturation of the flowing blood pool. We aimed to show that an adiabatic 90° excitation, long-TR, 7T ^31^P-CMRS protocol will reverse this balance, allowing robust cardiac pH_i_ measurements in healthy subjects and patients with hypertrophic cardiomyopathy (HCM).

**Methods:**

The cardiac Pi T_1_ was first measured by the dual TR technique in seven healthy subjects. Next, ten healthy subjects and three HCM patients were scanned with 7T ^31^P-MRS using long (6 s) TR protocol and adiabatic excitation. Spectra were fitted for cardiac metabolites including Pi.

**Results:**

The measured Pi T_1_ was 5.0 ± 0.3 s in myocardium and 6.4 ± 0.6 s in skeletal muscle. Myocardial pH was 7.12 ± 0.04 and Pi/PCr ratio was 0.11 ± 0.02. The coefficients of repeatability were 0.052 for pH and 0.027 for Pi/PCr quantification. The pH in HCM patients did not differ (*p* = 0.508) from volunteers. However, Pi/PCr was higher (0.24 ± 0.09 vs. 0.11 ± 0.02; *p* = 0.001); Pi/ATP was higher (0.44 ± 0.14 vs. 0.24 ± 0.05; *p* = 0.002); and PCr/ATP was lower (1.78 ± 0.07 vs. 2.10 ± 0.20; *p* = 0.020), in HCM patients, which is in agreement with previous reports.

**Conclusion:**

A 7T ^31^P-CMRS protocol with adiabatic 90° excitation and long (6 s) TR gives sufficient SNR for Pi and low enough blood signal to permit robust quantification of cardiac Pi and hence pH_i_. Pi was detectable in every subject scanned for this study, both in healthy subjects and HCM patients. Cardiac pH_i_ was unchanged in HCM patients, but both Pi/PCr and Pi/ATP increased that indicate an energetic impairment in HCM. This work provides a robust technique to quantify cardiac Pi and pH_i_.

## Background

Phosphorus cardiovascular magnetic resonance spectroscopy (^31^P-CMRS) is a valuable technique for non-invasive measurement of high-energy metabolites, e.g. adenosine triphosphate (ATP) and phosphocreatine (PCr), that allows assessment of tissue energy metabolism in vivo [1, 2]. The cardiac PCr/ATP ratio has been established as a biomarker that changes in most major cardiac disease states [3–5], and which predicts mortality in patients with dilated cardiomyopathy [3]. Decreased cardiac PCr/ATP ratios are also observed in obesity [6] and type-II diabetes [7], revealing cardiac involvement in these systemic diseases.

In principle, ^31^P-CMRS can also be used to measure cardiac intracellular pH (pH_i_) and the free energy change of ATP hydrolysis (ΔG_ATP_). However, these require accurate determination of the inorganic phosphate (Pi) signal frequency and amplitude. These are currently not robustly accessible because signals from 2,3-diphosphoglycerate (2,3-DPG) in the ventricular blood pools often obscure the signal from Pi in the myocardium.

Several groups reported pH_i_ and the Pi/PCr ratio measured in patients with hypertrophic cardiomyopathy (HCM) by ^1^H-decoupled ^31^P-CMRS at 1.5 T in the 1990s [8, 9]. In healthy subjects, the detection of cardiac Pi using ^1^H-decoupling was not possible in all subjects, primarily due to the small concentration of Pi in the heart and the inherently low signal-to-noise ratio (SNR) of ^31^P-CMRS at 1.5T [9]. Scanning at ultra-high field (7T) increases the SNR of cardiac ^31^P-CMRS substantially [10]. Additionally, it also enhances the spectral separation between signals from different metabolites. This may enable detection of cardiac Pi without ^1^H-decoupling, which is infeasible at 7T due to limits on specific absorption rate (SAR). However, typical ^31^P cardiac spectra are acquired using short TR of about 1 s, and low flip-angle to maximize the SNR/time [10]. Based on skeletal muscle data [11], it can be expected that cardiac Pi will have relatively long relaxation time T_1_ (several seconds), and thus, experience strong partial saturation. At the same time, the majority of blood in the ventricles is replenished at each heartbeat, so the undesired 2,3-DPG signal is undiminished when scanning with a surface radiofrequency (RF) coil, as is the case in almost all cardiac ^31^P-CMRS studies [12].

Furthermore, it is essential to know the excitation flip-angle for quantification of metabolite ratios, e.g., Pi/PCr. Regrettably, surface RF coils have a transmit field (B_1_^+^) that drops off rapidly with distance from the coil. This makes metabolite quantification difficult, especially at ultra-high fields. We recently demonstrated feasibility of excitation with a B_1_^+^-insensitive 90° adiabatic pulse for cardiac ^31^P-CMRS at 7T, which simplifies the quantification of metabolite ratios [13].

The aim of this work was to test the performance and robustness of a cardiac 7T ^31^P-CMRS protocol with a relatively long TR = 6 s and 90° adiabatic excitation for quantification of cardiac intracellular pH (pH_i_) and Pi/PCr ratio in the human heart. To this end, we also measured the T_1_ relaxation time of cardiac Pi using the dual TR technique [14] in seven healthy subjects; performed test-retest reproducibility of intracellular pH calculation in eight healthy subjects; and demonstrated our approach in three patients with HCM.

## Methods

**Fig. 1:**
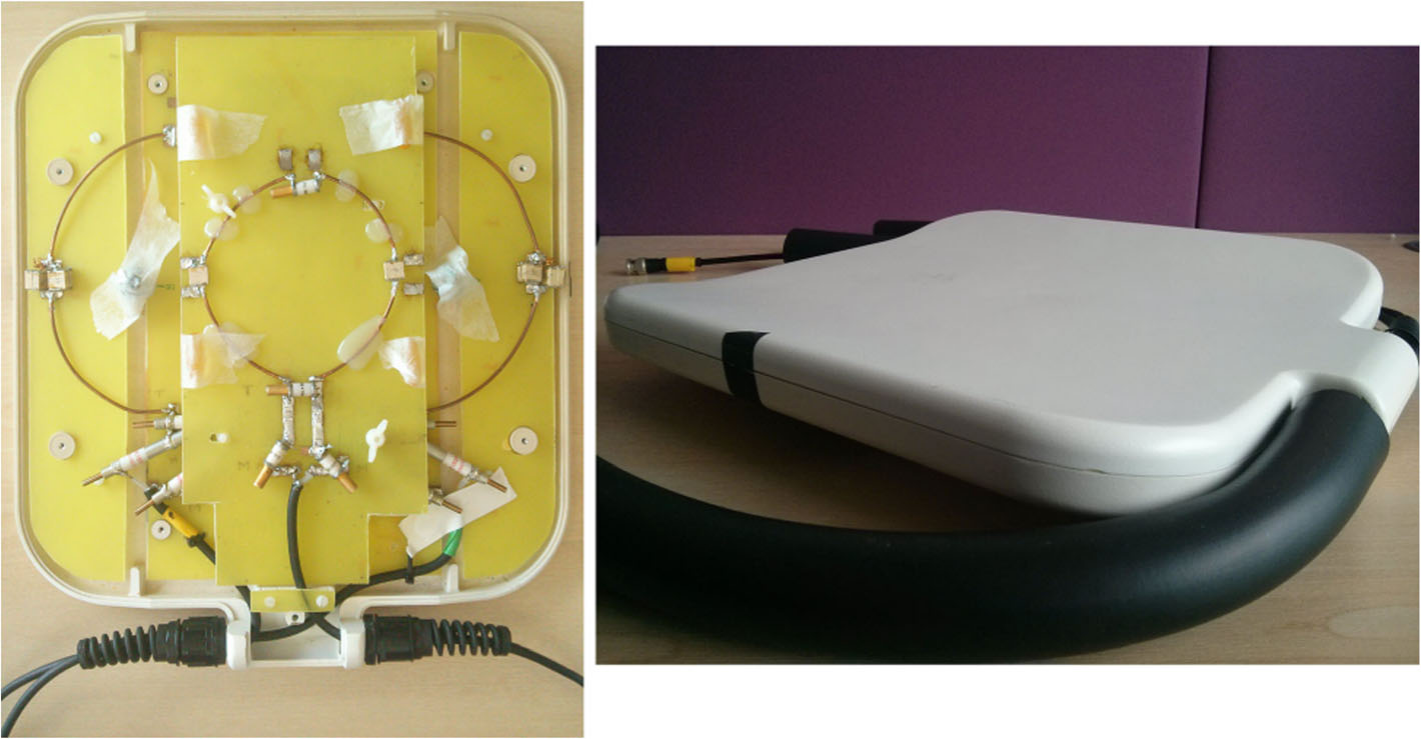
A photograph of our custom-built dual-tuned (^31^P/^1^H) surface RF-coil, comprising a quadrature ^31^P coil (two 15 cm loops, with overlap decoupling) and a single ^1^H loop (10 cm in diameter) described in detail in Schaller et al. [15]

All measurements were performed on a 7T whole body, research-only MR system (Siemens Healthineers, Erlangen, Germany) equipped with a custom-built dual-tuned (^31^P/^1^H) surface RF-coil, comprising a quadrature ^31^P coil (two 15 cm loops, with overlap decoupling) and a single ^1^H loop (10 cm in diameter) for localization (Fig. 1) [15]. The coil was positioned over the heart of participants lying supine. No cardiac gating or respiratory triggering were used [10].

### In vivo experiments

In total, 12 healthy subjects (27 ± 5 years; 3 females) and three HCM patients were recruited in compliance with local regulations and institutional ethics committee after giving informed consent. The in vivo experiments can be divided into three main groups: (a) direct comparison of short TR and long TR acquisition, using an amplitude modulated excitation pulse, or a B_1_^+^ insensitive adiabatic half-passage (AHP) pulse; (b) measurement of T_1_ of cardiac Pi; and (c) quantification of cardiac Pi/PCr and pH_i_ in healthy subjects and HCM patients. Some of the subjects took part in several of the in vivo experiments.

The feasibility to detect cardiac Pi using long vs short TR was first tested in one healthy subject by using two consecutive 3D ultrashort echo time (UTE) chemical shift imaging (CSI) [16] acquisitions. The first had a “short” (1 s) TR, and the other a “long” (5 s) TR. Both had matrix size (8 × 16 × 8), field of view (FOV) (240 × 240 × 200 mm^3^) and used acquisition weighting. This translates into an effective voxel size of 50.7mL [13]. The number of averages (NA) was matched for equal acquisition time, giving 41 averages for the short and 5 averages for the long TR measurement. An amplitude-modulated excitation pulse (1 ms long) applied in previous 7T studies was used [10].

Next, the recently proposed AHP pulse [13] was applied in three volunteers. In order to use this relatively SAR demanding pulse (7.5 ms long) with short TR (1 s), the 3D UTE-CSI [16] sequence was modified to allow an interleaved TR acquisition with two different TRs [17]. In this case, the second TR was matched to the effective TR in the AHP study, i.e. 6 s TR [13]. Matrix size and FOV matched the first experiment and acquisition weighting with 5 averages at the centre of k space was used for both TRs. The excitation frequency of the AHP pulse was centred at the 2,3-DPG signal, i.e. + 700 Hz relative to PCr.

**Fig. 2:**
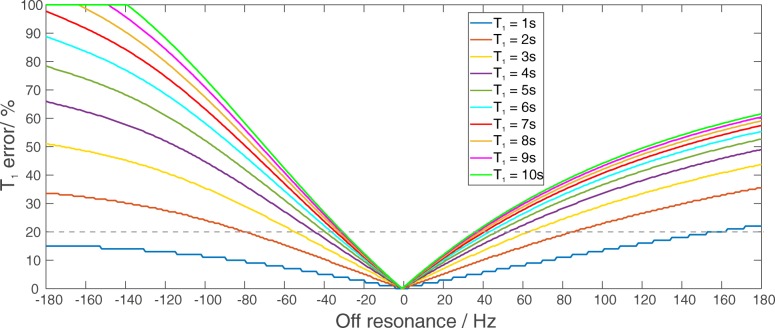
Simulations of the Bloch Equations for potential off-resonance errors in T_1_ measured using the dual TR technique with AHP excitation, at the lowest B_1_^+^ expected in vivo at the depth of the heart, i.e. ~ 10 cm from the coil. The simulations were performed for resonance offsets ranging from − 180 Hz to 180 Hz from the centre frequency and for the T_1_ values ranging from 1 s to 10 s. The 20% error margin for T_1_ estimation is depicted as a grey dashed line

Seven subjects were consecutively scanned using the dual TR technique [14] for determination of the T_1_ of cardiac Pi, which is required for quantification of the detected myocardial Pi. Since the interleaved TR acquisition is not suitable for T_1_ determination [17], two successive 3D UTE-CSI [16] acquisitions were required. The SAR limits for the narrow-banded AHP pulse [13] we used allowed for a 3 s minimum TR. Our chosen TR values were therefore: TR_1_ = 3 s and TR_2_ = 6 s. The matrix size and FOV again matched the original protocol, i.e. 8 × 16 × 8 and 240 × 240 × 200 mm^3^, respectively. The AHP pulse we used has a relatively narrow bandwidth for typical B_1_^+^ in the heart for our coil. So, the excitation was centred at + 600 Hz from PCr and the smooth side of the pulse’s frequency profile was facing towards PCr. Numerical solutions of the Bloch Equations were performed in Matlab (MathWorks, Natick, Massachusetts, USA) to estimate the potential errors in calculated T_1_ for resonance offsets from − 180 Hz to + 180 Hz from centre frequency, for the T_1_ values from 1 s to 10 s (Fig. 2).

Ten healthy subjects then underwent cardiac 7T ^31^P-CMRS examinations for cardiac pH, Pi/PCr and Pi/ATP quantification using interleaved AHP excitation [13]. In short, the AHP excitation was interleaved to excite the PCr and ATP region in odd acquisitions and the Pi and DPG region in even acquisitions. The effective TR was 6 s and other parameters matched the T_1_ measurements. The total acquisition time was 46 min and 42 s. In a subset of these subjects (*n* = 8), the measurement was performed twice to assess the repeatability of cardiac pH_i_ and Pi/PCr determination.

Finally, to demonstrate the feasibility of the technique in patients, three male patients with established diagnoses of HCM (57 ± 12 years, mean wall thickness 21 ± 2 mm) were recruited and scanned with the interleaved AHP protocol [13] to determine their cardiac pH, Pi/PCr and Pi/ATP. The acquisition time for patients was the same as for healthy subjects, i.e. 46 min and 42 s.

### Data analysis

For each subject, three septal voxels in the two mid left ventricular slices (six in total) were selected for further analysis. In addition, six chest muscle voxels were also selected for the T_1_ analysis. Spectra from all selected voxels were fitted using the OXSA toolbox [18] and its implementation of the AMARES time-domain fitting algorithm [19]. The PCr signal, fitted as single Lorentzian, and the γ-ATP signal, fitted as a Lorentzian doublet, were quantified from the odd acquisition spectra. The Pi and 2,3-DPG signals, all fitted as single Lorentzians, were quantified from the even acquisition spectra. The resonance frequency of Pi was constrained to the 4.7–5.1 ppm range and the two 2,3-DPG peaks were constrained to 5.2–5.7 ppm and 6.0–6.3 ppm, respectively. The same constraints for linewidth, i.e. 20-60 Hz, without dependencies on other peaks were applied for both 2,3-DPG signals and for Pi signal. If a limit was reached during fitting, fitting was repeated with the constraints extended by ±5 Hz.

The T_1_ relaxation of Pi was determined in cardiac and skeletal muscle voxels using the dual TR technique [14]. ATP and Pi signals were corrected for blood contamination as previously described [20]. Cramér-Rao lower bounds (CRLB) [21] were determined for the Pi peak amplitudes and voxels with the CRLB ≥15% were excluded. Remaining cardiac data were further corrected for partial saturation effects using the calculated T_1_ values for Pi and literature T_1_ values for γ-ATP and PCr [10]. As our voxels are relatively big, to confirm that the quantification of Pi did not depend on how much blood contamination was in the voxel, we have performed a correlation analysis between our reported blood-corrected Pi/PCr values and the 2,3-DPG/PCr ratio using all evaluated voxels, i.e. including the repeatability data. The cardiac intracellular pH_i_ was calculated from the chemical shift between Pi and PCr using the modified Henderson-Haselbach equation [22]. The Pi/PCr ratio and pH_i_ were compared between the two repeated measurements of healthy subjects by Bland-Altman analysis of agreement [23]. Coefficients of repeatability were estimated as 1.96 × √2 × intra-subject standard deviation (SD) [24]. Data are presented as mean ± SD. Student’s t-test was used to compare the PCr/ATP, Pi/ATP, Pi/PCr and pH_i_ of healthy subjects and HCM patients, with *p* < 0.05 considered statistically significant.

## Results

**Fig. 3:**
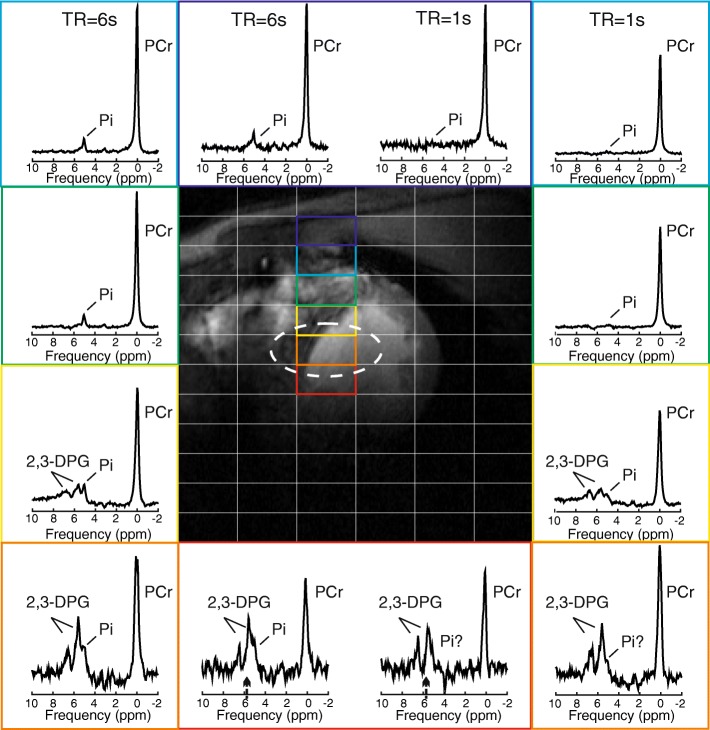
Spectra acquired with the interleaved TR sequence using a single AHP excitation frequency centred at the 2,3-DPG resonance (marked with a black arrow near the x-axis in the red voxel spectra). Spectra are shown for voxels in the chest wall muscle down to the intraventricular septum. Note the increased Pi amplitude in all voxels acquired at longer TR, demonstrating the improved detectability of Pi, which allows for reliable cardiac pH_i_ determination. The effective voxel size is depicted using a dashed white line

In the datasets acquired using the short TR, it was not possible to distinguish a Pi peak in voxels of interventricular septum, due to strong overlapping 2,3-DPG signals from blood. Although similarly strong 2,3-DPG signals were observed in the data acquired with long TR, the long TR acquisition allowed clear visibility of the Pi peak above 2,3-DPG in these voxels (Fig. 3).

**Fig. 4:**
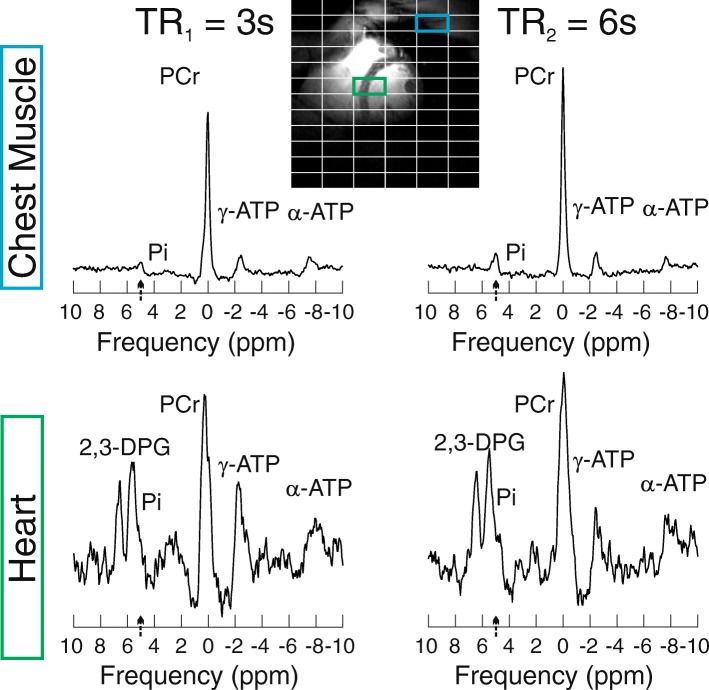
^31^P-CMRS spectra acquired in the chest muscle (blue voxel - upper row) and human heart (green voxel - bottom row) with TR_1_ = 3 s (left) and TR_2_ = 6 s (right). Spectra were acquired with a single AHP excitation frequency centred on Pi (marked with a black arrow near the x-axis). Please note that while spectra are scaled equally for TR_1_ and TR_2_, the heart spectra are scaled differently than the skeletal muscle spectra. A 30 Hz Lorentzian filter was applied to all spectra for better visualization. A pronounced Pi signal can be seen in TR_2_ spectra both in chest muscle and in the heart. Due to the limited excitation bandwidth of the AHP pulse, β-ATP peak is not excited, and thus, not shown

Our Bloch simulations showed that the error in T_1_ estimation, using the dual TR technique and selected TRs of 3 s and 6 s, increases with increasing frequency offset (Fig. 2). There is a steeper rise in T_1_ error on the sharper side of the AHP pulse frequency profile. Nevertheless, T_1_ errors < 20% are predicted for frequency offsets between − 30 Hz and + 35 Hz throughout the simulated T_1_ range (1–10 s). Representative spectra from skeletal muscle and interventricular septum are depicted in Fig. 4. The mean measured T_1_ relaxation time of Pi was 6.4 ± 0.6 s in chest muscle tissue and 5.0 ± 0.3 s in interventricular septum.

**Fig. 5:**
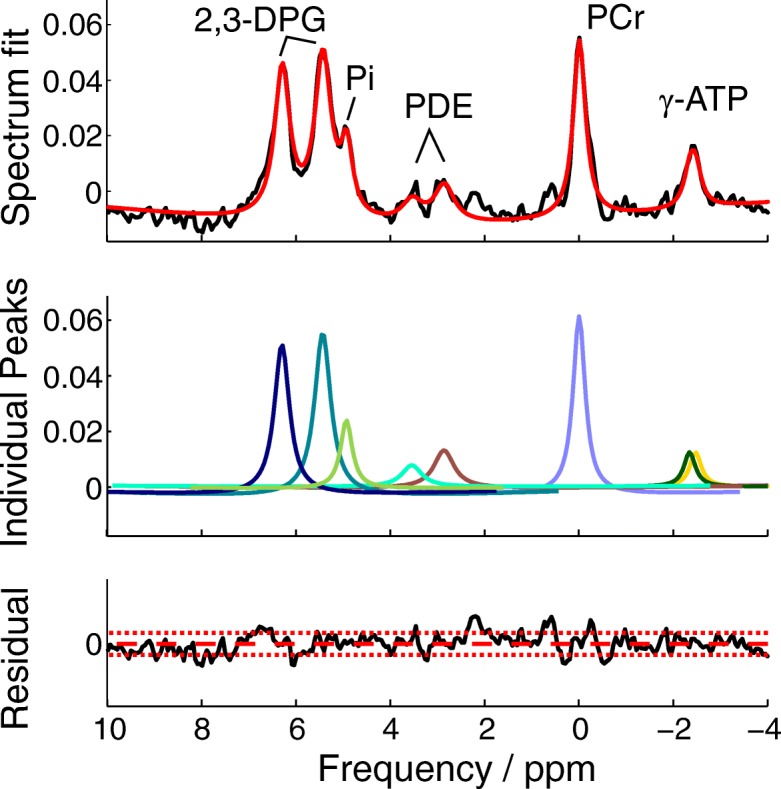
A representative result of the fitting procedure. A mid-septal spectrum measured during an even acquisition overlaid by the final spectral fit (top); individual peaks fitting (middle) and the residual (bottom) is depicted

In total, 108 septal voxels were analysed for pH and Pi quantification in healthy subjects, i.e. 6 voxels per measurement (*n* = 18, including the scan-rescan data). Out of these, 93 voxels had CRLB of Pi < 15%, with the mean CRLB of 10.6 ± 1.8%. On average, 5.2 ± 1.0 voxels per dataset were analysed further, with at least 4 voxels used in 17 datasets and 2 voxels in one. Figure 5 depicts a representative fit of a cardiac voxel acquired during the even acquisition.

**Fig. 6:**
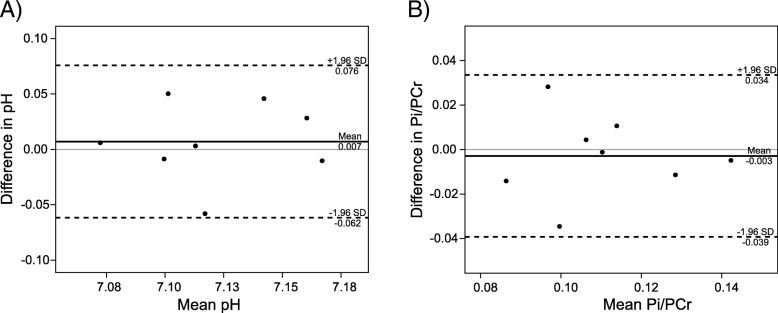
Bland-Altman plots of the in vivo test-retest quantification of pH (**a**) and Pi/PCr ratio (**b**) in the human heart septum using long TR ^31^P-CMRS with adiabatic excitation at 7 T. The mean absolute differences between the two scans were 0.007 for pH and 0.003 for Pi/PCr, showing very low bias. The estimated repeatability coefficients were 0.052 for pH and 0.027 for the Pi/PCr ratio

Table 1 denotes the calculated metabolite ratios and cardiac pH_i_ for each subject. The mean myocardial pH of healthy subjects was 7.12 ± 0.04 and the Pi/PCr ratio was 0.11 ± 0.02. Figure 6 depicts the Bland-Altman plots of the in vivo test-retest measurements of myocardial pH and Pi/PCr. The mean absolute biases given by the analysis of agreement were only 0.007 and 0.003, with the limits of agreement of ±0.069 and ± 0.036 for pH and Pi/PCr, respectively. The estimated repeatability coefficients were 0.052 and 0.027 for pH and Pi/PCr, respectively. No correlation was found between the blood-corrected Pi/PCr and 2,3-DPG/PCr values in this study (r = 0.001, *p* = 0.992).

**Table 1:** Calculated metabolite ratios and cardiac pH_i_ for every volunteer and patient

Participant	PCr/ATP		Pi/ATP		Pi/PCr		pH	
	Meas.1	Meas.2	Meas.1	Meas.2	Meas.1	Meas.2	Meas.1	Meas.2
Healthy subject #1	1.79 ± 0.39	1.86 ± 0.51	0.22 ± 0.19	0.24 ± 0.17	0.12 ± 0.10	0.13 ± 0.09	7.10 ± 0.13	7.10 ± 0.06
Healthy subject #2	2.15 ± 0.68	2.28 ± 1.09	0.25 ± 0.14	0.17 ± 0.10	0.11 ± 0.03	0.08 ± 0.04	7.08 ± 0.15	7.07 ± 0.07
Healthy subject #3	1.82 ± 0.38	2.04 ± 0.44	0.24 ± 0.08	0.29 ± 0.09	0.14 ± 0.07	0.14 ± 0.05	7.17 ± 0.28	7.15 ± 0.08
Healthy subject #4	2.25 ± 0.63	2.17 ± 0.63	0.24 ± 0.10	0.22 ± 0.12	0.11 ± 0.04	0.10 ± 0.07	7.16 ± 0.11	7.17 ± 0.04
Healthy subject #5	2.32 ± 0.47	2.37 ± 0.58	0.19 ± 0.10	0.23 ± 0.16	0.08 ± 0.04	0.12 ± 0.11	7.11 ± 0.05	7.11 ± 0.02
Healthy subject #6	2.08 ± 0.41	1.84 ± 0.45	0.17 ± 0.10	0.22 ± 0.05	0.08 ± 0.06	0.09 ± 0.03	7.09 ± 0.05	7.15 ± 0.02
Healthy subject #7	2.14 ± 0.62	2.10 ± 0.68	0.22 ± 0.22	0.23 ± 0.15	0.11 ± 0.12	0.11 ± 0.09	7.13 ± 0.03	7.08 ± 0.02
Healthy subject #8	2.19 ± 0.19	2.37 ± 0.43	0.27 ± 0.12	0.26 ± 0.12	0.12 ± 0.05	0.11 ± 0.05	7.17 ± 0.01	7.12 ± 0.02
Healthy subject #9	2.36 ± 0.53	N/A	0.35 ± 0.24	N/A	0.14 ± 0.10	N/A	7.10 ± 0.05	N/A
Healthy subject #10	1.93 ± 0.57	N/A	0.24 ± 0.11	N/A	0.13 ± 0.06	N/A	7.14 ± 0.06	N/A
Mean	2.10 ± 0.20	2.13 ± 0.21	0.24 ± 0.05	0.23 ± 0.04	0.11 ± 0.02	0.11 ± 0.02	7.12 ± 0.04	7.12 ± 0.04
HCM #1	1.75 ± 0.45		0.48 ± 0.28		0.26 ± 0.12		7.16 ± 0.03	
HCM #2	1.73 ± 0.67		0.56 ± 0.32		0.32 ± 0.16		7.12 ± 0.04	
HCM #3	1.86 ± 0.33		0.28 ± 0.14		0.14 ± 0.05		7.14 ± 0.07	
Mean	1.78 ± 0.07*		0.44 ± 0.14*		0.24 ± 0.09*		7.14 ± 0.02	
% change	−15		+ 83		+ 118		0.21	

Data are given as inter-voxel mean ± SD and the mean data are given as inter-subject mean ± SD, *denotes significant difference (*p* < 0.05) between healthy subjects and HCM patients

Values from both measurements of the repeatability study are given

**Fig. 7:**
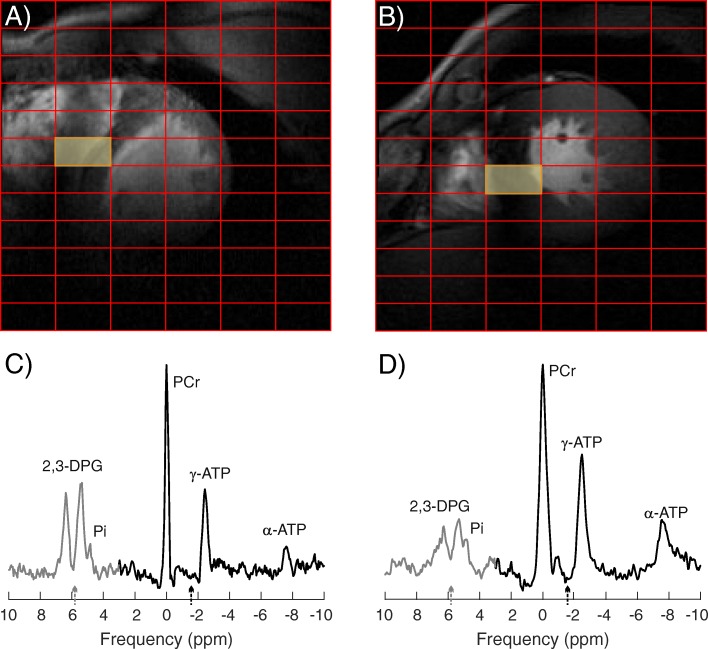
Representative mid-septal spectra with corresponding localizers acquired in a healthy subject (**a**, **c**) and an HCM patient (**b**, **d**). The spectra shown are a combination of the two interleaved AHP frequency data sets: one acquired with the excitation centred between PCr and γ-ATP (black) and the other with the excitation centred between the 2,3-DPG resonances (grey). The excitation frequencies are marked with arrows of corresponding colour near the x-axis. Spectra are scaled equally for the healthy subject and the HCM patient. Please note the more dominant cardiac Pi signal and ATP signals in the patient’s spectrum and the reduced 2,3-DPG reflecting that more of the voxel is filled with myocardium due to LV wall thickening

Figure 7 shows representative septal spectra acquired in a healthy subject and an HCM patient. More dominant cardiac Pi signal can be seen in the patient’s spectrum. A lower PCr/ATP ratio (1.78 ± 0.07 vs. 2.10 ± 0.20; *p* = 0.020) and higher Pi/ATP (0.44 ± 0.14 vs. 0.24 ± 0.05; *p* = 0.002) and Pi/PCr (0.24 ± 0.09 vs. 0.11 ± 0.02; *p* = 0.001) ratios were found in HCM patients compared to young healthy subjects. Detailed information is given in Table 1.

## Discussion

We proposed using 7T ^31^P-CMRS with adiabatic excitation and long TRs to quantify cardiac Pi and determine pH_i_ in vivo. Our results show that this approach allows robust detection of cardiac Pi in healthy subjects and also patients with HCM. The direct detection of cardiac Pi makes cardiac pH_i_ determination straightforward. We demonstrated excellent repeatability of this method, with limits of agreement: ±0.069 and ± 0.036 for pH and Pi/PCr, respectively. We also measured the T_1_ for myocardial Pi at 7T, which permits quantification of cardiac Pi/PCr.

Our protocol using long TR acquisition and adiabatic 90° excitation at 7T allowed for detection of myocardial Pi in vivo. In typical cardiac ^31^P-CMRS experiments, Pi is obscured by a dominant overlapping signal from 2,3-DPG in the blood pool. This approach is successful because the long TR reduces the effect of partial T_1_ saturation on Pi, which in typical short TR acquisitions strongly reduces the myocardial tissue signal leaving the 2,3-DPG signal from in-flowing blood almost intact. We demonstrated the effect of TR on Pi detection by comparing long and short TR acquisitions in the same subjects.

The majority of previously published studies quantifying cardiac pH_i_ by ^31^P-CMRS used ^1^H-decoupling to reduce the 2,3-DPG linewidth and minimise overlap with Pi [8, 9]. Although, ^1^H-decoupled 1.5 T ^31^P-CMRS was shown to be effective in HCM patients, its feasibility in healthy subjects was limited, e.g. de Roos et al. reported a 44% success rate (*n* = 4/9) in healthy subjects [9]. Jung et al. were able to reliably fit myocardial Pi in only 82% (*n* = 9/11) of cases [8]. In 100% of our healthy subject (*n* = 18/18), it was possible to find at least two septal voxels where AMARES fitted the Pi peak reliably (i.e. with CRLB < 15%), and in 94% cases we were able to fit Pi robustly in at least four out of our six targeted voxels. The improved SNR from scanning at 7T makes our approach robust. In future, we envisage that optimised per-subject B_0_ shimming would further aid separation of Pi and 2,3-DPG signals in our approach [25]. An alternative technique for determining cardiac pH_i_ was introduced by Blamire et al., who recorded spectra with and without saturation of the γ-ATP resonance. Subtracting these spectra suppresses 2,3-DPG and leaves signal from Pi generated by ATP hydrolysis [26]. They also achieved a 100% success rate (*n* = 6/6) for cardiac pH_i_ determination but unlike our method, their approach does not allow for direct Pi/PCr quantification because Pi is visible only in the difference spectrum [26].

We have measured the T_1_ of Pi in myocardial septum as well as in skeletal muscle, to allow quantification of Pi. This was possible using the dual TR technique [14]. Although the shortest possible TR was limited by SAR, our simulations show that our TRs would have < 20% error in T_1_. The method can tolerate 0.5 ppm mis-adjustment of the ^31^P transmitter frequency. The mean T_1_ value we measured in the chest muscle (6.4 ± 0.6 s) is in good agreement with literature [10, 11]. Our mean measured T_1_ value for cardiac Pi (5.0 ± 0.3 s) was shorter than the literature values measured in the calf muscle at 7T (6.3 ± 1.0 s [11] and 6.7 ± 0.2 s [10]). This is in agreement with shorter T_1_ times of PCr and ATP reported in the heart in comparison to skeletal muscle [10]. Our T_1_ of Pi measured in human heart is also in good accordance with the value reported for 2 T (4.3 ± 2.4 s) [27] given that T_1_ values change with field strength [11].

We found no significant difference in cardiac pH_i_ between healthy subjects and HCM patients (7.12 ± 0.04 vs 7.14 ± 0.02; *p* = 0.508). Even though our patient sample is rather small, this agrees with previous reports [8, 9, 28]. Our measured cardiac pH_i_ agrees with literature values showing mean cardiac pH_i_ ranging from 7.06 to 7.15 for healthy subjects [8, 9, 26, 28, 29]. Furthermore, our repeatability test showed good agreement between the cardiac pH_i_ measured in the two acquisitions, with the limits of agreement being 0.007 ± 0.069 and the estimated repeatability coefficient being 0.052. This is to our knowledge the first published reproducibility for cardiac pH_i_ measurement published.

Previous studies have reported both a reduced PCr/ATP and an increased Pi/PCr in HCM patients compared to healthy subjects [8, 9, 28, 30]. In line with these findings, we have found a lower PCr/ATP ratio in HCM patients (1.78 ± 0.07 vs. 2.10 ± 0.20; − 15%; *p* = 0.020). Furthermore, Pi/PCr (0.24 ± 0.09 vs. 0.11 ± 0.02; 118%; *p* = 0.001) and Pi/ATP (0.44 ± 0.14 vs. 0.24 ± 0.05; 83%; *p* = 0.002) ratios were significantly higher in HCM compared to controls. Comparing absolute numbers, de Roos et al. [9] reported Pi/PCr ratios of 0.14 ± 0.06 and 0.20 ± 0.04 for healthy subjects and HCM patients, i.e. a 43% increase. Similarly, Jung et al. [8] reported 0.10 ± 0.07 and 0.20 ± 0.08 Pi/PCr ratios in healthy control subjects and HCM patients, i.e. an increase of 100%. We observed a slightly larger (118%) increase of Pi/PCr ratios in HCM patients, albeit in a small cohort. Our Bland-Altman analysis of agreement between repeated measurements of Pi/PCr ratio showed limits of agreement of − 0.003 ± 0.036 and the estimated repeatability coefficient was 0.027. It has been proposed previously that increased Pi in the heart may directly inhibit contractile function since the release of Pi from the actin-myosin cross bridge is associated with force development [31] and was shown to correlate with the myocardial contractile force at the onset of ischemia in animal studies [32]. It has been also suggested lately that the Pi is both the primary feedback signal for stimulating oxidative phosphorylation in vivo and also the most significant product of ATP hydrolysis in limiting the capacity of the heart to hydrolyse ATP in vivo [33]. The scope of this technical work was limited, and thus at this time we can neither confirm nor refute these previous findings on the changes in Pi and pH_i_ due to HCM. However, this work paves the way for studies that will be able to test these theories in more adequately powered larger patient cohorts.

The main limitation of our study is the relatively long acquisition time required (47 min) due to the combination of 3D-CSI localisation and long 6 s TR. However, trying to shorten the scan time by reducing the TR would limit the detectability of cardiac Pi, and it would also likely exceed SAR restrictions because of the adiabatic excitation pulses used [13]. Faster localization strategies, e.g., 1D-image selected in vivo spectroscopy (1D-ISIS) or 1D-CSI could potentially be combined with our approach for cardiac Pi detection, but our RF-coil has insufficient peak B_1_^+^ for an effective inversion pulse and too large a sensitive volume for effective localisation of cardiac signal by 1D-ISIS or 1D-CSI [13]. Acquisition of data with higher spatial resolution, i.e. smaller voxel size, could also help to minimize the signal contamination from the blood pool, but this again requires comparably long acquisition time even with a short TR and reduced number of averages, which could in turn limit the detectability of cardiac Pi in healthy subjects [34].

## Conclusion

In conclusion, we present a technique for reliable quantitation of cardiac Pi and determination of cardiac intracellular pH (pH_i_) using 7T ^31^P-CMRS with adiabatic excitation and a long TR. We achieved 100% success rate for Pi detection in the hearts of 10 consecutively recruited healthy subjects and in three HCM patients. We were also able to measure the longitudinal relaxation time of cardiac Pi at 7T. This allowed for quantification of the cardiac Pi/PCr and Pi/ATP ratios, which were significantly increased in three patients with HCM. This new technique will allow addressing an array of clinically relevant questions related to the role of Pi in cardiac diseases.

## Abbreviations

1D-ISIS: 1D-image selected in vivo spectroscopy
2,3-DPG: 2,3-diphosphoglycerate
^31^P-CMRS: Phosphorus cardiovascular magnetic resonance spectroscopy
AHP: Adiabatic half-passage
ATP: Adenosine triphosphate
CRLB: Cramér-Rao lower bounds
CSI: Chemical shift imaging
FOV: Field of view
HCM: Hypertrophic cardiomyopathy
NA: Number of averages
PCr: Phosphocreatine
Pi: Inorganic phosphate
RF: Radiofrequency
SAR: Specific absorption rate
SD: Standard deviation
SNR: Signal-to-noise ratio
TR: Repetition time
UTE-CSI: Ultrashort echo time chemical shift imaging
